# A review of American pharmacy: education, training, technology, and practice

**DOI:** 10.1186/s40780-016-0066-3

**Published:** 2016-11-09

**Authors:** Scott J. Knoer, Allison R. Eck, Amber J. Lucas

**Affiliations:** 1 Cleveland Clinic, 9500 Euclid Avenue, Cleveland, OH 44195 USA; 2 Cleveland Clinic Main Campus, 9500 Euclid Avenue, Cleveland, OH 44195 USA; 3 Obstetrics and Neonatology, Olathe Medical Center, 20333 West 151st Street, Olathe, KS 66062 USA

**Keywords:** Pharmacist education, Pharmacist training, Professional practice, Practice model, Technology, Pharmacy enterprise

## Abstract

In the United States, pharmacists are responsible for the provision of safe, effective, efficient, and accountable medication related-care for hospital and health-system patients. Leveraging automated technologies, pharmacy technicians, and pharmacist extenders are the means through which efficient, effective, and safe medication use processes are created and maintained. These strategies limit the amount of pharmacist resources needed for nonjudgmental tasks such as medication distribution, allowing more capacity for advanced direct patient care roles.

Pharmacists are directly integrated into interprofessional medical teams. Pharmacists optimize patient outcomes through a variety of channels, including: providing recommendations for evidence-based medication selection on patient care rounds; offering drug information to other health care providers and patients; monitoring therapeutic responses; and reconciling medications as patients transition across the continuum of care.

Achieving the highest level of pharmacy practice necessitates that United States pharmacists are soundly educated and trained. Pharmacist education, training, and professional practice models closely mirror those of physicians. Many health-systems also pursue credentialing and privileging of pharmacists to ensure competency and facilitate growth and development. Advanced training, along with credentialing, privileging, and collaborative practice agreements have positioned pharmacists to serve as stewards of the medication use system, champions of patient safety, and essential contributors to optimal patient outcomes.

## Background

The United States’ (U.S.) healthcare system has undergone significant reform in the 21^st^ century. Focus on cost-effective care in pay-for-performance models has accelerated the need for hospitals and health-systems to achieve positive patient outcomes through leveraging the most appropriate resources. This shift is reflected in the practice of pharmacy with enhanced emphasis on pharmacists managing medication use across the continuum of patient care.

The American Society of Health-System Pharmacists (ASHP) Pharmacy Practice Model Summit of 2011 stated that “pharmacists are responsible for the provision of safe, effective, efficient, and accountable medication related-care for hospital and health-system patients” [1]. Leveraging automated technologies, pharmacy technicians, and pharmacist extenders are the means through which efficient, effective, and safe medication use processes are created and maintained. These strategies limit the amount of pharmacist resources needed for nonjudgmental tasks such as medication distribution, allowing more capacity for advanced direct patient care roles.

Achieving the highest level of pharmacy practice necessitates that U.S. pharmacists are soundly educated through Doctor of Pharmacy, residency, and certification programs. Many health-systems also pursue credentialing and privileging of pharmacists to ensure competency and facilitate growth and development. Advanced training, along with credentialing, privileging, and collaborative practice agreements have positioned pharmacists to serve as stewards of the medication use system, champions of patient safety, and essential contributors to optimal patient outcomes.

## Main text

### Education and training

#### Pharmacy education

Pharmacist education, training, credentialing, and professional practice models closely mirror those of physicians in the U.S. In parallel to the physicians’ clinical Doctor of Medicine (M.D.) degree, pharmacists complete a university clinical degree program at the Doctor of Pharmacy (Pharm.D.) level. In 2000, the Pharm.D. became the exclusive entry-level degree conferred upon graduating pharmacists. The Pharm.D. is a four year professional degree program completed after a minimum of two years of pre-requisite undergraduate coursework. However, some U.S. colleges of pharmacy require an additional level of coursework and completion of a baccalaureate degree prior to admission to pharmacy school.

The Pharm.D. curriculum consists of didactic and experiential education that meets the standards set by the Accreditation Council for Pharmacy Education (ACPE). ACPE released new curriculum standards in July 2016. This was a result of ongoing efforts to incorporate advances in real-world pharmacy practice with more focus on patient-centered care, interprofessional teams, evidence-based practice, quality improvement, and informatics [2].

The didactic curriculum of Pharm.D. education includes a foundation of pharmacy science courses such as pharmacokinetics, pharmacology, medicinal chemistry, and pharmacotherapy. Additional didactic curriculum is also taught in areas such as medication safety, pharmacy law and ethics, biostatistics, toxicology, epidemiology, hands-on skill-based labs, evidence-based practice, innovation, and business management.

The practical curriculum incorporates multiple experiential elements. These include the newly added Interprofessional Education (IPE) requirement, as well as the traditional Introductory Pharmacy Practice Experiences (IPPE) and Advanced Pharmacy Practice Experiences (APPE). IPE experiences incorporate pharmacy student interactions with other healthcare providers and students in simulations or real-time scenarios such as patient rounds with the medical team. IPPE rotations are two to four week experiences in both community and hospital pharmacy settings scheduled in the middle of the didactic curriculum. These rotations help students grow by applying knowledge in different areas of practice.

APPE rotations are four to six weeks in length and are scheduled after the completion of didactic training in the last year of the Pharm.D. curriculum. Goals of APPE rotations include: 1) focusing the majority of time on the provision of direct patient care, 2) gaining experience treating diverse patient populations, 3) engaging in collaborative patient-care decision-making with members of an interprofessional healthcare team, 4) demonstrating competence in community, ambulatory care, health-system pharmacy, and general medicine, and 5) allowing students to explore areas of interest and gain exposure to professional opportunities [2].

Successful completion of both the didactic and practical pharmacy curriculum prepares a student to apply for pharmacist licensure.

#### Pharmacist licensure

Pharmacist licensure and re-licensure is governed at the state level by individual Boards of Pharmacy. Licensure indicates that state requirements to practice pharmacy are met. Candidates for licensure in all states must pass the North American Pharmacist Licensure Examination (NAPLEX). This competency-based examination applies knowledge gained in pharmacy education to real-life practice situations. All states also require a law examination incorporating both federal and state laws. Most utilize the Multistate Pharmacy Jurisprudence Examination (MPJE) adapted with state-specific laws, rules, and regulations as they apply within that state’s Pharmacy Practice Act. Re-licensure by state boards of pharmacy occurs after pharmacists complete ongoing requirements, which usually include a specific number of hours and type of continuing education and verification that the pharmacist is in good legal standing.

Traditional pharmacy professional development in the U.S. has consisted of continuing education hours required by most states to maintain licensure [3]. As the profession becomes increasingly complex, ongoing education is shifting to a more robust, continuous professional development (CPD) model. CPD is defined as an ongoing self-directed, structured, outcomes-focused cycle of learning and personal improvement [4].

As scope of pharmacy practice evolves with more complex roles and responsibilities in patient care, the traditional model of learning is changing with it. To ensure quality patient care, advanced training and certifications that are voluntarily achieved by pharmacists are starting to become required in many practice settings. Newer models of competency-based education incorporate application of knowledge and demonstration of skills in both controlled and real-life situations [5].

#### Post-graduate training

Post-graduate residency training, similar to physician residency training, is becoming a required credential for entry-level health-system pharmacy practice. More specialized clinical practice positions may require additional specialty residency training. Completion of an accredited residency program is a credential that differentiates pharmacists from the general requirements for licensure. A Post-Graduate Year One (PGY-1) residency program is the baseline of residency training. It is a 12 month organized, directed, accredited program that builds upon knowledge, skills, attitudes and abilities gained from pharmacy school [6]. It is designed to enhance general competencies in managing medication use systems and supports optimal medication therapy outcomes for patients with a broad range of disease states.

A Post-Graduate Year Two (PGY-2) residency program is also 12 months in length and builds on the competencies achieved in a PGY-1 residency [7]. It is often referred to as a specialty residency as its focus is within a specific area of pharmacy practice, such as oncology, pediatrics, ambulatory care, or management. A PGY-2 residency increases the depth of knowledge related to medication therapy and clinical leadership in the specific area of focus. Graduates of a PGY-2 program are prepared to pursue Board Certification if it exists in that specialty.

Fellowships are directed, highly-individualized, postgraduate programs designed to prepare the participant to become an independent researcher. Fewer of these opportunities exist compared to residency programs. This is often the path pursued for new graduates interested in practicing in the pharmaceutical industry or academia.

#### Advanced certification

Board certification through the Board of Pharmaceutical Specialties (BPS) is a credential required or preferred in advanced practice settings. Certification for pharmacists is in alignment with physician certification through the American Board of Medical Specialties [8]. The BPS has recognized several domains of critical skills necessary for advanced practice in different disease states and patient populations [9]. By developing validated certification exams, this credential has become the gold standard of advance practice qualification. For each recognized specialty, BPS forms a Specialty Council of content experts and psychometric consultants to develop test questions with real-life practice relevance. Once certification is achieved through the exam process, knowledge and skills are maintained through recertification every seven years.

BPS certifications for pharmacists today include ambulatory care, critical care, nuclear pharmacy, nutrition support, geriatrics, oncology, pediatrics, pharmacotherapy, and psychiatric pharmacy. Added qualifications are available in infectious disease and cardiology.

Pharmacists can also acquire other less intensive certifications and certificate programs that are available to multiple disciplines and not pharmacist-specific. These certifications are typically received after a short focused training course and are available in specialty areas such as anticoagulation, asthma, diabetes, pain management, sterile product preparation, and advanced cardiac life support.

Post-graduate residency training in a program accredited by ASHP and specialty certification through the BPS are supported by several professional pharmacy organizations [10, 11]. ASHP’s accreditation program and the BPS serve as the most recognized advanced pharmacist training and competence assessment platforms in the U.S.

### Practice model

#### Distribution models

Healthcare reform in the U.S. has accelerated the mergers of smaller hospitals into large complicated multi-hospital health-systems. These unions are created so that organizations can achieve operational and clinical efficiencies of scale [12]. Today’s health-systems often include a broad array of services in both the inpatient (acute hospital) and outpatient (ambulatory or clinic) environment. Pharmacy must connect the dots of these disparate segments and oversee medication use across all sectors of patient care. Optimizing patient outcomes and financial success by managing all aspects of the medication use continuum in an organization is referred to as leading the pharmacy enterprise [13].

In the U.S., pharmacy practice is rooted in safe, efficient medication distribution models. In hospitals, intravenous medications are compounded centrally in pharmacies in accordance with United States Pharmacopeia (USP) 797 guidelines. In July 2018, the compounding and handling of hazardous medications will be similarly regulated by USP chapter 800. USP 797 and 800 promote best practices for safety in sterile and hazardous medication compounding and are legally required by most state boards of pharmacy [14].

Technology and automation are used throughout the medication use process in healthcare facilities. Medications are barcoded and barcode scanning is used for inventory stocking, dose preparation, repackaging, dispensing, and administration. Barcode technology enhances patient safety and the quality of care by improving accuracy and limiting human error [15, 16]. In addition to barcode scanning, automation such as dispensing and IV robots, carousels, automated dispensing cabinets, and IV compounding software contribute to safe and efficient medication distribution.

#### Pharmacy technicians

Well-educated and highly skilled pharmacy technicians are an integral part of hospitals and health-systems. Technicians perform most non-judgmental tasks under the direction of a pharmacist, such as preparing, compounding, and delivering medication; and managing pharmacy automation. Advanced pharmacy technicians perform additional tasks such as obtaining patients’ medication histories, inventory management, quality improvement initiatives, and working in advanced medication systems including tech-check-tech models [17].

Although pharmacy technicians are certified by a national Pharmacy Technician Certification Board (PTCB), the requirement for registration or licensure and the scope of their responsibilities are set by individual state laws, which vary widely. Joint efforts by the National Association of Boards of Pharmacy (NABP), ACPE, and ASHP will elevate training and certification standards for pharmacy technicians at a national level. Beginning in 2020, PTCB will require new candidates for certification to complete an ASHP/ACPE-accredited pharmacy technician education program [18]. Driving toward a national standard for pharmacy technician education, certification, and registration/licensure will expand the scope of technician practice, allowing for further reallocation of pharmacist resources to direct patient care and advanced practice [19, 20].

#### Pharmacist extenders

Training of student and resident pharmacists is an important responsibility of health-system pharmacists. As greater emphasis is placed on providing cost-effective services, extra activities assumed by pharmacy must be associated with measurable improvements in patient outcomes. Leveraging pharmacist extenders creates opportunity for expanded direct patient care activities in a manner that is cost-effective while contributing to meaningful student and resident learning experiences. This is commonly implemented through a layered learner model [21].

The layered learner model consists of pharmacists precepting pharmacy residents, who teach and precept pharmacy students to deliver direct patient care beyond the reach of what the pharmacist could achieve alone [22]. The layered learner model allows for pharmacy services such as medication histories and reconciliation, facilitating bedside delivery of discharge medications, and patient education on new and high risk medications. These increased opportunities for patient interaction contribute to a higher quality of patient care for a greater number of patients and lead to increased patient satisfaction [23].

#### Clinical initiatives

In hospitals, pharmacists are directly integrated into interprofessional medical teams. Pharmacists optimize patient outcomes through a variety of channels, including: providing recommendations for evidence-based medication selection on patient care rounds; offering drug information to other health care providers and patients; monitoring therapeutic responses; and reconciling medications as patients transition across the continuum of care [24].

Pharmacists also direct medication use in health-systems through involvement in pharmacy and therapeutics (P&T) committees. The P&T Committee is a medical and pharmacy group that is responsible for selecting and managing the formulary, which is a list of institutionally approved medications available in a hospital. This committee oversees policies governing the use of medications through the development of medication guidelines, order sets, and care pathways. The P&T committee may also supervise the credentialing and privileging of the pharmacists employed by the health-system [25].

#### Credentialing and privileging

Credentialing has been used in the medical profession for many years and is gaining recognition and acceptance in pharmacy. Credentialing is a process used by organizations to validate professional licensure, clinical experience and other requirements for a specialized practice. It consists of documentation of qualifications that are expected of a healthcare provider to practice in a specific setting [26]. As scope of pharmacy practice evolves, the credentialing process must adapt to verify a pharmacist’s ongoing competence to provide specific pharmacy services. Competence assessment is an ongoing process. Credentialing of pharmacists to establish competence and privileges for advanced practice is currently the responsibility of the organization the individual works for. Professional pharmacy organizations and the Council on Credentialing in Pharmacy are working to establish guidance and standards for advanced clinical pharmacy practice [27]. It is anticipated that the latter will serve to guide credentialing committees to better understand pharmacy’s credentials and the systems by which credentials are issued.

Privileging, which is used extensively for physicians, is the process that defines a specific service provided by a healthcare practitioner. It ensures the individuals being granted privileges within a healthcare organization are competent and capable of performing certain activities [26].

### Future direction

#### Population health

Due to historical financial payment models and incentives, the U.S. healthcare system has focused primarily on providing acute care instead of wellness and preventative care. The American healthcare system is the best in the world for treating complex medical conditions, such as uncontrolled diabetes and mental health diagnoses [28, 29], and performing cutting-edge surgical processes, such as face transplants [30]. However, the U.S. lags many developed countries in terms of population health management (PHM). Population health is defined in the American Journal of Public Health as, “the health outcomes of a group of individuals, including the distribution of such outcomes within the group” [31]. Due to its historical lack of focus on PHM, the U.S. trails many developed countries in patient outcomes including infant mortality, immunization status, morbidity from chronic diseases, and overall life expectancy [32].

As a result of the unsustainable trajectory of healthcare spending in the U.S., payment models are moving toward a more population health focused system. As health-systems are financially incented to keep patients healthy, organizations are transitioning to models more adept at PHM. Pharmacists, and their ability to impact cost and outcomes through effectively managing the medication continuum across levels and sites of care, are critical to the success of the healthcare system [33].

#### Collaborative practice

In order for our organizations to succeed in global payment, “at risk” and PHM models, all caregivers in the health-system must be utilized to their full potential in a cost-effective strategy. Ensuring that healthcare providers are optimized in a multi-disciplinary care team is referred to as practicing at the “top of license.” Physicians must focus on tasks requiring their unique skills including diagnosing patients and leading the rest of the care-team. Nurses must focus on providing nursing care, dieticians must administer to a patient’s nutritional needs and social workers must address the psychosocial needs of the patient.

Pharmacists in the U.S. are increasingly recognized by a broad array of stakeholders as the medication use experts in Patient Centered Medical Home and other interdisciplinary care team models [34–39]. In order to maximize patient outcomes, pharmacists must accept accountability for managing medication use across the care continuum. Once the physician diagnoses the patient, the pharmacist is increasingly responsible for medication selection, patient education, monitoring, and modifying drug therapy. Pharmacists are best able to manage patient’s medication therapy through collaborative practice agreements.

Collaborative practice is loosely defined as a pharmacist or group of pharmacists entering into an agreement with a physician or group of physicians which allows the pharmacist to manage patient medication therapy on the physician’s behalf. Pharmacists’ ability to practice within these agreements varies widely from state to state [40].

As mentioned previously, pharmacy practice is governed at the state level. The most advanced pharmacy practice occurs in states with liberal collaborative practice laws. In these states, pharmacists can add, modify, and discontinue medications and order appropriate laboratory tests.

#### Provider status, a financial barrier to pharmacist expansion

While pharmacists are advancing their ability to legally practice at a high level state by state, one of the biggest barriers to aggressive expansion of pharmacy services in the ambulatory clinic domain is the inability of pharmacists to charge federal healthcare programs (Medicare and Medicaid) for their services. Pharmacists are not recognized as “providers” under Section 1861 of the Social Security Act [41]. Not being listed among accepted practitioners such as physicians, nurse practitioners, and physician assistants limits pharmacists’ ability to generate revenue for the services they provide.

Because nurse practitioners and physician assistants are recognized and can bill for services, they are much more numerous in outpatient settings than are pharmacists. ASHP and the American Pharmacists Association are actively pursuing provider status through the federal legislative process.

#### Extended training and ever-evolving opportunities

A PGY-1 pharmacy residency is seen as the baseline for working within the acute hospital and ambulatory clinic setting. Completing a PGY-2 specialty residency and achieving board certification is increasingly becoming a requirement for working in specialized areas. As more pharmacists achieve advanced training and as our patient care becomes even more complicated, there is growing debate around the potential need for PGY-3 residencies in sub-specialties which would mimic the advancement of physician training. Potential sub-specialties include advanced heart failure and cardiac transplantation, allergy / immunology, bone marrow transplant, endocrine, diabetes and metabolism, gastroenterology, maternal-fetal medicine, medical toxicology, neonatology, neurology and pediatric hematology / oncology [42].

## Conclusions

The U.S. system for educating, training, licensing, certifying, credentialing, and continually assessing pharmacists has undergone dramatic changes over the last twenty years. A key driver of this increased level of educational sophistication was the requirement of the all Pharm.D. curriculum. The expansion of and move toward requiring residency training and board certification has continued to advance the level of care that pharmacists provide in the U.S.

Evolution of payment models that reward cost effective resource utilization as opposed to over-treatment provides opportunities for pharmacists to positively impact the healthcare system and to improve population health.

As enabling Collaborative Practice Acts and provider status recognition progress, pharmacists will make an increasingly positive contribution to the health of the patients and communities they serve.

## Abbreviations

ACPE: Accreditation council for pharmacy education
APPE: Advanced pharmacy practice experiences
ASHP: American society of health-system pharmacists
BPS: Board of pharmaceutical specialties
CPD: Continuous professional development
IPE: Interprofessional education
IPPE: Introductory pharmacy practice experiences
M.D.: Doctor of medicine
MPJE: Multistate pharmacy jurisprudence examination
NABP: National association of boards of pharmacy
NAPLEX: North American pharmacist licensure examination
P&T: Pharmacy and therapeutics
PGY-1: Post-graduate year one
PGY-2: Post-graduate year two
Pharm.D.: Doctor of Pharmacy
PHM: Population health management
PTCB: Pharmacy technician certification board
U.S.: United States
USP: United States pharmacopeia
